# Anti-Inflammatory, Anti-Obesity, and Insulin-Sensitizing Effects of *Chamaecrista nomame* (Siebold) H. Ohashi Extract in Cellular Models, Including TNF-α-Induced Adipocyte Dysfunction

**DOI:** 10.3390/foods15111858

**Published:** 2026-05-24

**Authors:** Min-Hye Kim, Ji-Hyun Im, Xiaolu Fu, June-Seok Lim, Je-Won Park, MinWoo Baek, Ok-Hwan Lee

**Affiliations:** 1 Department of Food Biotechnology and Environmental Science, Kangwon National University, Chuncheon 24341, Republic of Korea; minhye8733@naver.com (M.-H.K.); ijh108020@gmail.com (J.-H.I.); fuxiaolu2019@gmail.com (X.F.); dlawnstjr725@naver.com (J.-S.L.); jw_park1998@naver.com (J.-W.P.); 2 Agriculture and Life Science Research Institute, Kangwon National University, Chuncheon 24341, Republic of Korea

**Keywords:** *Chamaecrista nomame* (Siebold) H. Ohashi, luteolin, anti-inflammatory, anti-obesity, insulin resistance, TNF-α

## Abstract

*Chamaecrista nomame* (Siebold) H. Ohashi (*C. nomame*), a leguminous plant traditionally consumed in East Asia, contains diverse bioactive phytochemicals, but whether its activities act convergently under obesity-related pathological conditions remains unclear. This study investigated the anti-inflammatory, anti-obesity, and insulin-sensitizing effects of a 40% ethanol extract of *C. nomame* (ECNE) and its marker compound luteolin in lipopolysaccharide (LPS)-stimulated RAW 264.7 macrophages, differentiating and mature 3T3-L1 adipocytes, and tumor necrosis factor-α (TNF-α)-induced insulin-resistant adipocytes. In LPS-stimulated macrophages, ECNE and luteolin reduced nitric oxide and pro-inflammatory cytokine (TNF-α, interleukin (IL)-6, IL-1β) production, accompanied by suppression of nuclear factor-κB and mitogen-activated protein kinase signaling. In differentiating adipocytes, both reduced lipid accumulation and downregulated peroxisome proliferator-activated receptor γ, CCAAT/enhancer-binding protein α, and adipocyte protein 2. In mature adipocytes, they enhanced insulin-stimulated glucose uptake and Akt phosphorylation. In TNF-α-challenged adipocytes, pretreatment partially restored glucose uptake and Akt phosphorylation while attenuating IL-6 and monocyte chemoattractant protein-1 production. ECNE exerted effects equal to or greater than those of luteolin at equivalent luteolin-based concentrations, indicating contributions from additional phenolic constituents. These findings support ECNE as a multifunctional natural resource against obesity-associated inflammation and insulin resistance.

## 1. Introduction

Obesity is a major global health concern characterized by excessive fat accumulation and is closely associated with metabolic disorders such as insulin resistance, type 2 diabetes, and cardiovascular diseases [1]. In particular, chronic low-grade inflammation in adipose tissue has been recognized as a key contributor to obesity-associated metabolic dysfunction [2]. Hypertrophic adipocytes secrete increased levels of pro-inflammatory cytokines and chemokines, including interleukin (IL)-6 and monocyte chemoattractant protein-1 (MCP-1), which promote macrophage infiltration and establish a persistent inflammatory microenvironment [3,4]. This inflammatory state disrupts insulin signaling pathways and ultimately impairs glucose homeostasis [5]. Among these mediators, tumor necrosis factor-α (TNF-α) plays a central role in linking inflammation to insulin resistance. TNF-α is overexpressed in obese adipose tissue and induces the production of pro-inflammatory cytokines, while simultaneously interfering with insulin signaling, resulting in reduced insulin responsiveness in adipocytes [6,7]. In this context, inflammation- and metabolism-related signaling pathways represent converging features of disease progression rather than isolated events [8]. However, to elucidate their regulation, it is first necessary to determine whether natural compounds can independently modulate key inflammatory and metabolic signaling pathways before addressing their integrated effects under coexisting pathological conditions. *Chamaecrista nomame* (Siebold) H. Ohashi (*C. nomame*), a leguminous plant traditionally consumed in East Asia, contains various bioactive phytochemicals, including flavonoids such as luteolin [9,10]. Previous studies have suggested its antioxidant, anti-inflammatory, and anti-obesity potential [11,12]; however, these bioactivities have been examined in isolated cellular systems, and whether they act convergently under pathological conditions that coexist in obese adipose tissue remains unclear. Because inflammation and insulin resistance occur simultaneously rather than independently in obese adipose tissue, a TNF-α-induced adipocyte dysfunction model that recapitulates both inflammatory activation and impaired insulin responsiveness provides a physiologically relevant in vitro platform to address this question [13,14]. Furthermore, although luteolin itself has been extensively reported to exhibit anti-inflammatory and metabolic regulatory activities [15,16], it remains unclear whether the biological effects of *C. nomame* extract can be explained solely by its luteolin content or whether additional phenolic constituents contribute to its overall bioactivity under obesity-associated pathological conditions. Therefore, the present study first evaluated the effects of the 40% ethanol extract of *C. nomame* (ECNE) and its marker compound luteolin in three cellular models: lipopolysaccharide (LPS)-stimulated RAW 264.7 macrophages, differentiating 3T3-L1 adipocytes, and mature 3T3-L1 adipocytes. Their protective effects were then examined in TNF-α-treated mature 3T3-L1 adipocytes as a model of coexisting inflammation and insulin resistance. In addition, the effects of ECNE were compared with those of luteolin at equivalent luteolin-based concentrations to assess whether additional phenolic constituents contribute to the overall bioactivity of ECNE.

## 2. Materials and Methods

### 2.1. Chemicals and Reagents

Luteolin, LPS, rosiglitazone (RGZ), insulin, dexamethasone (DEX), 3-isobutyl-1-methylxanthine (IBMX), Oil Red O (ORO), and bovine serum albumin (BSA) were all sourced from Sigma-Aldrich (St. Louis, MO, USA). Recombinant murine TNF-α was procured from MedChemExpress (Monmouth Junction, NJ, USA). For cell culture, high-glucose Dulbecco’s modified Eagle’s medium (DMEM), fetal bovine serum (FBS), bovine serum (BS), penicillin–streptomycin (P/S), phosphate-buffered saline (PBS), and ethylenediaminetetraacetic acid (EDTA) were acquired from Gibco (Gaithersburg, MD, USA). A cell viability assay kit based on 2,3-bis(2-methoxy-4-nitro-5-sulfophenyl)-2H-tetrazolium-5-carboxanilide (XTT) was provided by WelGene (Seoul, Republic of Korea). Enzyme-linked immunosorbent assay (ELISA) kits specific for TNF-α, IL-6, interleukin-1β (IL-1β), and MCP-1 were obtained from R&D Systems (Minneapolis, MN, USA). Reagents for Western blot analysis included an enhanced chemiluminescence (ECL) detection reagent from Cytiva (Marlborough, MA, USA), along with Tris buffer, Tween 20, and a Bradford-based protein quantification reagent from Bio-Rad Laboratories (Hercules, CA, USA). Primary antibodies targeting inhibitor of nuclear factor-κB α (IκBα), nuclear factor-κB (NF-κB) p65, phospho-NF-κB p65, c-Jun N-terminal kinase (JNK), phospho-JNK, extracellular signal-regulated kinase (ERK)1/2, phospho-ERK1/2, p38 mitogen-activated protein kinase (MAPK), phospho-p38 MAPK, peroxisome proliferator-activated receptor γ (PPARγ), CCAAT/enhancer-binding protein α (C/EBPα), adipocyte protein 2 (aP2), protein kinase B (Akt), and phospho-Akt, together with their matching secondary antibodies, were supplied by Cell Signaling Technology (Danvers, MA, USA).

### 2.2. Preparation of ECNE

Dried *C. nomame* leaves were obtained from Gangwon Medicinal Herbs (Wonju, Republic of Korea). The leaves were pulverized, sieved through a 40-mesh screen, and kept at 4 °C prior to extraction. ECNE was prepared following the procedure described by Lim et al. [10] with slight modification. In previous studies on *C. nomame* extracts, 40% ethanol extraction yielded higher total phenolic content and stronger antioxidant activity compared with water extraction [10,11]. A similar trend has been reported in other plant materials, where 40% ethanol was identified as one of the most effective concentrations for phenolic compound extraction [17]. Based on these findings, 40% ethanol was selected as the extraction solvent in the present study. Fifty grams of the powdered leaves were subjected to reflux extraction with 500 mL of 40% aqueous ethanol at 80 °C for 2 h. After passing through Whatman No. 2 filter paper, the extract was evaporated to dryness at 40 °C using a rotary vacuum evaporator under reduced pressure and then freeze-dried to yield a powdered form of ECNE, which was kept at −20 °C until analysis. Quantification of luteolin in ECNE was performed by HPLC following the method reported by Lim et al. [10]; the luteolin content was 2.60 ± 0.01 mg/g of dried extract (mean ± SD, n = 3). A representative chromatogram is provided in Supplementary Figure S1. Based on this luteolin content and the molecular weight of luteolin (286.24 g/mol), the luteolin-equivalent concentrations corresponding to 100 and 300 μg/mL ECNE were calculated as approximately 0.9 and 2.7 μM, respectively. These luteolin concentrations were used as direct comparators in all cellular experiments to enable a one-to-one comparison between ECNE and its marker compound at equivalent luteolin-based levels.

### 2.3. Cell Culture and Treatment

#### 2.3.1. RAW 264.7 Macrophage Culture

The murine macrophage cell line RAW 264.7 was purchased from the American Type Culture Collection (ATCC, Manassas, VA, USA). Routine culture was carried out in DMEM containing 10% (*v*/*v*) FBS and 1% (*v*/*v*) P/S under standard conditions (37 °C, 5% CO_2_, humidified atmosphere). For experiments, a suspension of 1 × 10^5^ cells/well was dispensed into culture plates and incubated for 24 h to allow attachment. Cells were then pretreated with the indicated concentrations of ECNE or luteolin for 3 h, and subsequently challenged with LPS (0.1 μg/mL) for an additional 24 h. Upon completion of treatment, culture supernatants and cell lysates were harvested for downstream analyses.

#### 2.3.2. 3T3-L1 Preadipocyte Culture and Differentiation

3T3-L1 preadipocytes were purchased from the American Type Culture Collection (ATCC, Manassas, VA, USA). Preadipocytes were maintained in DMEM containing 10% (*v*/*v*) BS and 1% (*v*/*v*) P/S until full confluence was reached. The time point two days after confluence was designated as day 0 (D0). Adipogenic differentiation was initiated at D0 by switching the medium to DMEM formulated with 1 μg/mL insulin, 1 μM DEX, 0.5 mM IBMX, 10% FBS, and 1% P/S, which was maintained for 2 days (D0–D2).

For the differentiation experiment, the induction medium was replaced at D2 with DMEM supplemented with 10% FBS, 1% P/S, and 1 μg/mL insulin, and this medium was refreshed every two days until D6. ECNE or luteolin was introduced at D0 and maintained throughout the entire differentiation period, and the cells were collected at D6 for downstream analyses.

To generate fully matured adipocytes, cells were cultured in DMEM containing 10% FBS, 1% P/S, and 1 μg/mL insulin from D2 to D5; thereafter, the medium was changed to DMEM with 10% FBS and 1% P/S (without insulin) from D5 to D9 to allow complete maturation. Fully matured adipocytes at D9 were used for subsequent assays on mature adipocytes and for the TNF-α-induced adipocyte dysfunction model described in Section 2.3.3.

#### 2.3.3. TNF-α-Induced Adipocyte Dysfunction Model

Fully matured 3T3-L1 adipocytes at D9 (generated as described in Section 2.3.2) were used to establish a TNF-α-induced adipocyte dysfunction model that recapitulates both inflammatory activation and impaired insulin responsiveness. Cells were first exposed to ECNE or luteolin for 24 h, after which TNF-α was applied and incubation was continued for an additional 24 h. The treated cells were then subjected to the respective assays as described in the corresponding sections.

### 2.4. Cell Viability Assay

Cytotoxicity was examined with a commercial XTT-based kit (WelGene, Seoul, Republic of Korea) following the supplier’s protocol. RAW 264.7 macrophages and 3T3-L1 preadipocytes were plated in 96-well plates at 1 × 10^5^ and 2 × 10^6^ cells/well, respectively, and cultured as outlined in Section 2.3.1 and Section 2.3.2. Fully matured adipocytes (D9) used for TNF-α-related assays were prepared according to Section 2.3.2 and Section 2.3.3. After the indicated treatments, a working reagent mixture was prepared by combining the XTT solution with the PMS activator at a 50:1 (*v*/*v*) ratio, and 40 μL of this mixture was dispensed into each well. Plates were then kept at 37 °C under 5% CO_2_ for 4 h in a humidified incubator, and the optical density was recorded at 450 nm, with 690 nm as the reference wavelength, using a microplate reader (SpectraMax i3, Molecular Devices, Sunnyvale, CA, USA). Cell viability was expressed as a percentage of the untreated control.

### 2.5. Nitric Oxide (NO) Production

Nitrite levels in the culture medium, as an indicator of nitric oxide (NO) production, were determined using the Griess reaction. Following LPS stimulation as described above, culture supernatants were collected from treated RAW 264.7 cells. A volume of 100 μL of each supernatant was mixed with an equal volume of Griess reagent, which was freshly prepared by combining 1% (*w*/*v*) sulfanilamide in 5% phosphoric acid and 0.1% (*w*/*v*) N-(1-naphthyl)ethylenediamine dihydrochloride at a 1:1 ratio. The reaction mixture was incubated at room temperature for 10 min to allow color development, and absorbance was measured at 550 nm using a microplate reader. Nitrite concentrations were determined using a standard curve generated with sodium nitrite.

### 2.6. Cytokine Production

Secreted cytokines were quantified in culture supernatants by sandwich ELISA. After the indicated treatments, supernatants from LPS-stimulated RAW 264.7 macrophages or from TNF-α-challenged mature 3T3-L1 adipocytes were harvested and clarified by centrifugation (2000× *g*, 20 min, 4 °C). TNF-α, IL-6, and IL-1β were measured in the RAW 264.7 samples, whereas IL-6 and MCP-1 were measured in the 3T3-L1 samples, using commercial ELISA kits (R&D Systems, Minneapolis, MN, USA) in accordance with the protocols supplied by the manufacturer.

### 2.7. Oil Red O Staining

Intracellular lipid accumulation in differentiated 3T3-L1 adipocytes was visualized and quantified by ORO staining. On day 6 of differentiation, the culture medium was aspirated, and the cells were rinsed twice with 1× PBS. Fixation was performed in 10% (*v*/*v*) formalin for 1 h at ambient temperature, after which the fixed monolayers were briefly washed with 60% (*v*/*v*) isopropanol and allowed to dry at room temperature. The dried cells were then incubated for 1 h with a working ORO solution, which was freshly prepared by mixing the stock ORO solution and distilled water at a 6:4 (*v*/*v*) ratio. The wells were rinsed with distilled water three times and air-dried. For quantification, the retained dye was extracted with 100% isopropanol and transferred into a 96-well plate; optical density was then recorded at 490 nm using a microplate reader.

### 2.8. 2-NBDG Glucose Uptake Assay

Glucose uptake was assessed using the fluorescent glucose analog 2-[N-(7-nitrobenz-2-oxa-1,3-diazol-4-yl)amino]-2-deoxy-D-glucose (2-NBDG), adapted from a previously reported protocol [18]. Mature 3T3-L1 adipocytes were grown in black 96-well plates, serum-starved in DMEM without FBS for 2 h, and subsequently treated as described above. For acute insulin stimulation, insulin (100 nM) was added 30 min before the end of treatment. Cells were then rinsed with serum-free DMEM and incubated with 20 μM 2-NBDG for 30 min while protected from light. After removal of unincorporated probe by a PBS wash, fluorescence intensity was recorded on a microplate reader using excitation and emission wavelengths of 485 and 535 nm, respectively. Cells were seeded at comparable densities based on comparable confluence prior to the assay. The 2-NBDG fluorescence values were not additionally normalized to total protein content or cell number per well. However, XTT cell viability assays conducted under the same experimental conditions confirmed that neither ECNE/luteolin treatment nor TNF-α exposure significantly affected cell viability, and obvious cell detachment was not observed microscopically during the experiments. Therefore, the observed changes in 2-NBDG fluorescence are unlikely to be primarily explained by variations in cell number. Nevertheless, normalization to total protein content or cell number will be incorporated in future studies to further strengthen the quantitative assessment of glucose uptake activity.

### 2.9. Western Blot Analysis

After the indicated treatments, cells were washed with ice-cold PBS and lysed using a buffer containing protease and phosphatase inhibitors. For analyses of acute insulin signaling, insulin (100 nM) was applied 15 min prior to harvest. Lysates were clarified by centrifugation at 12,000× *g* for 15 min at 4 °C, and total protein concentrations were determined using the Bradford assay (Bio-Rad Laboratories, Hercules, CA, USA). Equal amounts of protein were separated on 10% SDS–PAGE gels and transferred onto PVDF membranes. Membranes were blocked for 1 h at room temperature with 5% (*w*/*v*) BSA in TBST. The membranes were incubated with the appropriate primary antibodies overnight at 4 °C, followed by incubation with horseradish peroxidase (HRP)-conjugated secondary antibodies for 1 h at room temperature. Immunoreactive bands were visualized using an ECL detection system and captured with a ChemiDoc imaging system (Bio-Rad Laboratories, Hercules, CA, USA). Band intensities were quantified by densitometric analysis using ImageJ software (version 1.53e, National Institutes of Health, Bethesda, MD, USA), and normalized to β-actin as a loading control.

### 2.10. Statistical Analysis

All quantitative data are presented as the mean ± standard deviation (SD) of at least three independent biological replicates (n = 3), each conducted on a separate day using independently passaged cells. Within each biological replicate, technical replicates were included to ensure measurement reliability, and the averaged technical replicate values were used for statistical analysis. Group differences were assessed by one-way analysis of variance (ANOVA), followed by Duncan’s multiple range test. All statistical analyses were performed with SPSS software (version 24.0; IBM Corp., Armonk, NY, USA), and a *p*-value below 0.05 was regarded as statistically significant.

## 3. Results and Discussion

### 3.1. Anti-Inflammatory Effects of ECNE and Luteolin in LPS-Stimulated RAW 264.7 Macrophages

#### 3.1.1. Cell Viability and NO Production

To evaluate the anti-inflammatory effects of ECNE and luteolin in LPS-stimulated RAW 264.7 macrophages, cell viability and NO production were examined.

**Figure 1:**
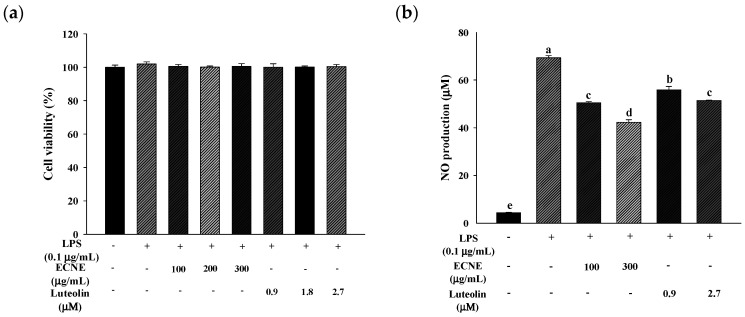
Effects of ECNE and luteolin on cell viability and NO production in LPS-stimulated RAW 264.7 macrophages. (**a**) Cell viability. (**b**) NO production. Data are presented as the mean ± SD of three independent experiments. Different lowercase letters indicate statistically significant differences among groups (*p* < 0.05) by Duncan’s multiple range test.

Cell viability was assessed using the XTT assay, which measures metabolically active cells based on the conversion of tetrazolium salt into a water-soluble formazan product by mitochondrial dehydrogenases [19]. As shown in Figure 1a, treatment with ECNE or luteolin did not induce cytotoxicity at any of the tested concentrations in LPS-stimulated RAW 264.7 cells. Based on these findings, two representative concentrations were selected for subsequent experiments: a low dose (100 μg/mL ECNE; 0.9 μM luteolin) and a high dose (300 μg/mL ECNE; 2.7 μM luteolin).

NO is a short-lived free radical synthesized from L-arginine by nitric oxide synthase (NOS). While NO functions as a signaling molecule under physiological conditions [20], its excessive production by inducible NOS (iNOS) during LPS-induced inflammation contributes to oxidative stress and promotes the release of pro-inflammatory cytokines [21]. Because NO is unstable, its production was indirectly assessed by measuring nitrite levels using the Griess reaction. As shown in Figure 1b, LPS stimulation markedly increased nitrite levels compared with the unstimulated control. Pretreatment with ECNE significantly attenuated this increase in a concentration-dependent manner, with reductions of approximately 27% and 39% at 100 and 300 μg/mL, respectively. Luteolin also significantly inhibited nitrite production, reducing levels by approximately 19% and 26% at 0.9 and 2.7 μM, respectively. Similar inhibitory effects of luteolin on NO production have been reported in macrophage models [22].

Notably, ECNE exerted a stronger inhibitory effect than luteolin alone at the corresponding luteolin-equivalent concentrations, suggesting that multiple phenolic constituents in ECNE, including luteolin, collectively contribute to its anti-inflammatory activity.

#### 3.1.2. Cytokine Production

**Figure 2:**
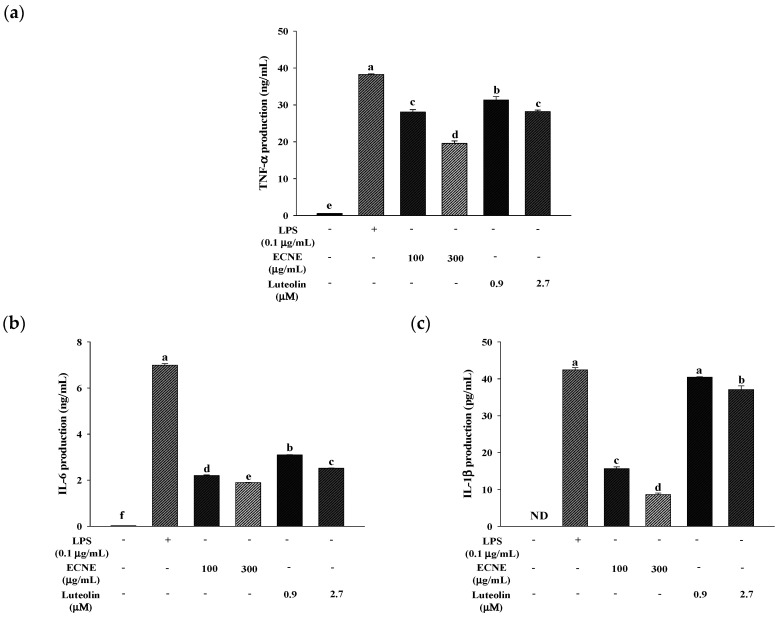
Effects of ECNE and luteolin on pro-inflammatory cytokine production in LPS-stimulated RAW 264.7 macrophages. (**a**) TNF-α. (**b**) IL-6. (**c**) IL-1β. Data are presented as the mean ± SD of three independent experiments. Different lowercase letters indicate statistically significant differences among groups (*p* < 0.05) by Duncan’s multiple range test. ND, not detected.

Pro-inflammatory cytokine secretion was next measured in LPS-stimulated RAW 264.7 macrophages. LPS stimulation markedly increased the production of TNF-α, IL-6, and IL-1β compared with the unstimulated control (Figure 2).

Pretreatment with ECNE significantly reduced cytokine secretion in a concentration-dependent manner. At 300 μg/mL, ECNE decreased TNF-α, IL-6, and IL-1β levels by approximately 49%, 80%, and 73%, respectively, relative to the LPS-treated group. At 100 μg/mL, reductions of approximately 27%, 63%, and 69% were observed. Luteolin also significantly attenuated cytokine production, reducing TNF-α, IL-6, and IL-1β levels by approximately 26%, 13%, and 64% at 2.7 μM, and 18%, 5%, and 56% at 0.9 μM, respectively. TNF-α, IL-6, and IL-1β are central mediators of macrophage-driven inflammation and contribute to the initiation and amplification of inflammatory responses [23]. The simultaneous reduction in these cytokines indicates that ECNE and luteolin attenuate the inflammatory response in LPS-activated macrophages. Consistent with previous reports, luteolin suppressed pro-inflammatory cytokine production in macrophage models [22]. Consistent with the NO results, ECNE exerted a stronger inhibitory effect than luteolin at the corresponding luteolin-equivalent concentrations.

#### 3.1.3. NF-κB Signaling

NF-κB is a key regulator of pro-inflammatory gene expression and is closely associated with the cytokine responses described in the previous section. To examine whether ECNE and luteolin modulate this pathway, IκBα and NF-κB p65 phosphorylation were analyzed by Western blotting in LPS-stimulated RAW 264.7 macrophages.

**Figure 3:**
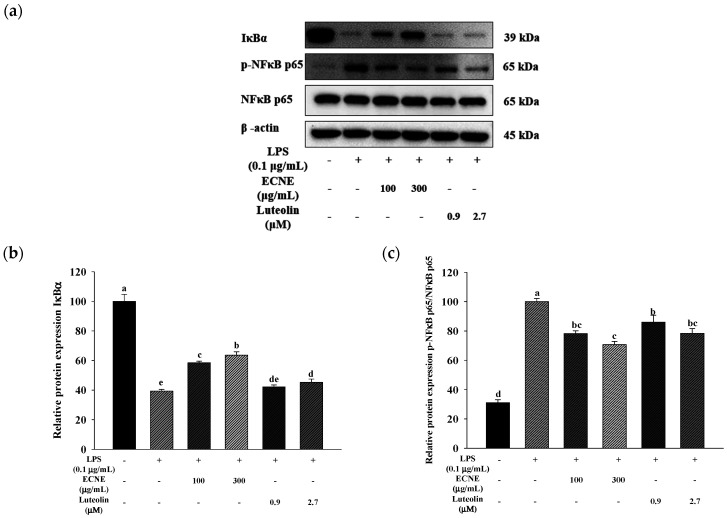
Effects of ECNE and luteolin on NF-κB signaling in LPS-stimulated RAW 264.7 macrophages. (**a**) Representative Western blot images of IκBα, phospho-NF-κB p65 (p-NF-κB p65), total NF-κB p65, and β-actin. (**b**) Relative protein expression of IκBα normalized to β-actin. (**c**) Relative protein expression of p-NF-κB p65 normalized to total NF-κB p65. Data are presented as the mean ± SD of three independent experiments. Different lowercase letters indicate statistically significant differences among groups (*p* < 0.05) by Duncan’s multiple range test.

Under basal conditions, NF-κB is sequestered in the cytoplasm by its inhibitor IκBα [24]. Inflammatory stimuli such as LPS induce IκBα degradation, thereby releasing NF-κB and allowing its nuclear translocation, which promotes the transcription of pro-inflammatory genes including TNF-α, IL-6, and IL-1β [25]. As shown in Figure 3a, LPS treatment markedly reduced IκBα expression and increased phosphorylation of p65 relative to the unstimulated control, while total p65 levels remained unchanged, consistent with canonical NF-κB activation. Pretreatment with ECNE attenuated these LPS-induced changes in a concentration-dependent manner, restoring IκBα expression and reducing p65 phosphorylation, with a more pronounced effect at 300 μg/mL than at 100 μg/mL. Quantitative densitometry confirmed that ECNE significantly restored IκBα expression (Figure 3b) and decreased the p-p65/p65 ratio (Figure 3c) relative to the LPS-treated group. Luteolin treatment showed similar trends, attenuating p65 phosphorylation and partially restoring IκBα.

These findings indicate that ECNE and luteolin suppress NF-κB activation by stabilizing cytoplasmic IκBα and limiting p65 phosphorylation, providing a molecular basis for the reduced TNF-α, IL-6, and IL-1β secretion observed in Figure 2. ECNE again exhibited a stronger inhibitory effect than luteolin at corresponding luteolin-equivalent concentrations.

#### 3.1.4. MAPK Signaling

**Figure 4:**
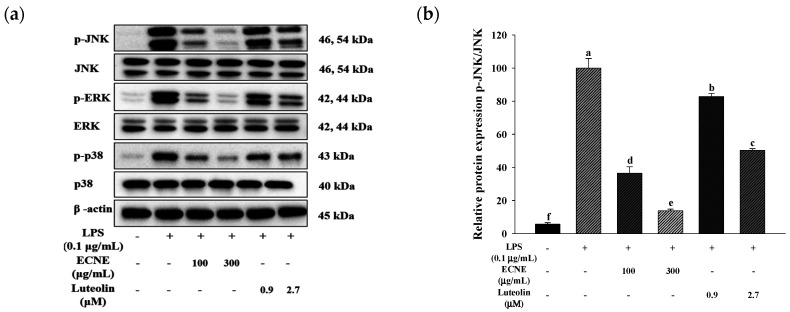

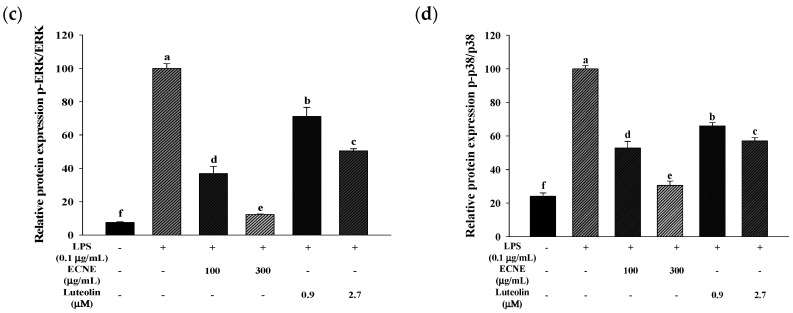
Effects of ECNE and luteolin on MAPK signaling in LPS-stimulated RAW 264.7 macrophages. (**a**) Representative Western blot images of phospho-JNK (p-JNK), JNK, phospho-ERK (p-ERK), ERK, phospho-p38 (p-p38), p38, and β-actin. (**b**) Relative protein expression of p-JNK normalized to total JNK. (**c**) Relative protein expression of p-ERK normalized to total ERK. (**d**) Relative protein expression of p-p38 normalized to total p38. Data are presented as the mean ± SD of three independent experiments. Different lowercase letters indicate statistically significant differences among groups (*p* < 0.05) by Duncan’s multiple range test.

In addition to NF-κB, the mitogen-activated protein kinase (MAPK) pathway also contributes to the regulation of pro-inflammatory gene expression in activated macrophages [26]. To determine whether ECNE and luteolin modulate this pathway, the phosphorylation of JNK, ERK, and p38 was assessed by Western blotting in LPS-stimulated RAW 264.7 macrophages. As shown in Figure 4a, basal phosphorylation levels of JNK, ERK, and p38 were low in unstimulated cells but were markedly increased following LPS stimulation, consistent with MAPK activation. Pretreatment with ECNE significantly attenuated LPS-induced phosphorylation of all three MAPKs in a concentration-dependent manner, while total JNK, ERK, and p38 protein levels remained comparable across all groups. Quantitative densitometry confirmed that ECNE significantly reduced the p-JNK/JNK (Figure 4b), p-ERK/ERK (Figure 4c), and p-p38/p38 (Figure 4d) ratios relative to the LPS-treated group. Luteolin also significantly decreased the phosphorylation of all three MAPKs, with a weaker effect than ECNE at corresponding luteolin-equivalent concentrations. The MAPK pathway is known to regulate the expression of pro-inflammatory mediators and to function in coordination with NF-κB signaling during inflammatory responses [27]. Therefore, the observed inhibition of JNK, ERK, and p38 phosphorylation by ECNE and luteolin suggests suppression of upstream signaling events that drive cytokine production in LPS-stimulated macrophages. Together with the inhibition of NF-κB activation shown in Figure 3, these findings indicate that ECNE exerts anti-inflammatory effects through coordinated modulation of multiple signaling pathways rather than a single molecular target. However, it should be noted that a pharmacological anti-inflammatory positive control (e.g., dexamethasone or indomethacin) was not included in the LPS-stimulated RAW 264.7 experiments. The inclusion of established anti-inflammatory drugs as positive controls will be considered in future studies to further contextualize the magnitude of the observed effects.

### 3.2. Anti-Obesity Effects of ECNE and Luteolin in Differentiating 3T3-L1 Adipocytes

#### 3.2.1. Cell Viability and Lipid Accumulation

**Figure 5:**
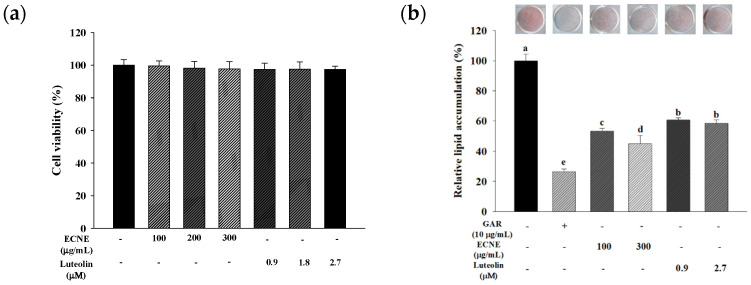
Effects of ECNE and luteolin on cell viability and lipid accumulation in 3T3-L1 adipocytes. (**a**) Cell viability assessed by the XTT assay. (**b**) Intracellular lipid accumulation visualized by Oil Red O staining (top panel) and quantified spectrophotometrically (bottom panel). *Garcinia cambogia* extract (GAR; 10 μg/mL) was used as a positive control. Data are presented as the mean ± SD of three independent experiments. Different lowercase letters indicate statistically significant differences among groups (*p* < 0.05) by Duncan’s multiple range test.

The anti-obesity potential of ECNE and luteolin was evaluated in differentiating 3T3-L1 adipocytes. Cell viability was assessed using the XTT assay. As shown in Figure 5a, neither ECNE nor luteolin affected cell viability relative to the untreated control, indicating that subsequent changes in adipogenesis were not attributable to cytotoxicity. Based on these results, 100 and 300 μg/mL ECNE and 0.9 and 2.7 μM luteolin were selected for further experiments. Intracellular lipid accumulation was visualized and quantified by Oil Red O (ORO) staining on day 6 of differentiation. As shown in Figure 5b, differentiated control cells exhibited abundant intracellular lipid droplets, whereas treatment with *Garcinia cambogia* extract (GAR), used as a positive control, markedly reduced lipid accumulation. Quantitative analysis revealed that ECNE significantly suppressed lipid accumulation in a concentration-dependent manner, reducing relative lipid levels to approximately 53% and 45% of the differentiated control at 100 and 300 μg/mL, respectively. Luteolin also reduced lipid accumulation, with relative levels of approximately 61% and 58% at 0.9 and 2.7 μM, respectively.

These findings indicate that ECNE and luteolin attenuate adipocyte differentiation, as reflected by reduced intracellular lipid accumulation. Given that lipid accumulation is a hallmark of adipocyte differentiation regulated by key transcription factors such as peroxisome proliferator-activated receptor γ (PPARγ) and CCAAT/enhancer-binding protein α (C/EBPα), the observed reduction suggests that ECNE may interfere with adipogenic regulatory pathways. ECNE again exhibited a stronger inhibitory effect than luteolin at corresponding luteolin-equivalent concentrations, implying that multiple phenolic constituents in ECNE, including luteolin, contribute to the inhibition of adipogenesis.

#### 3.2.2. Adipogenic Transcription Factors

**Figure 6:**
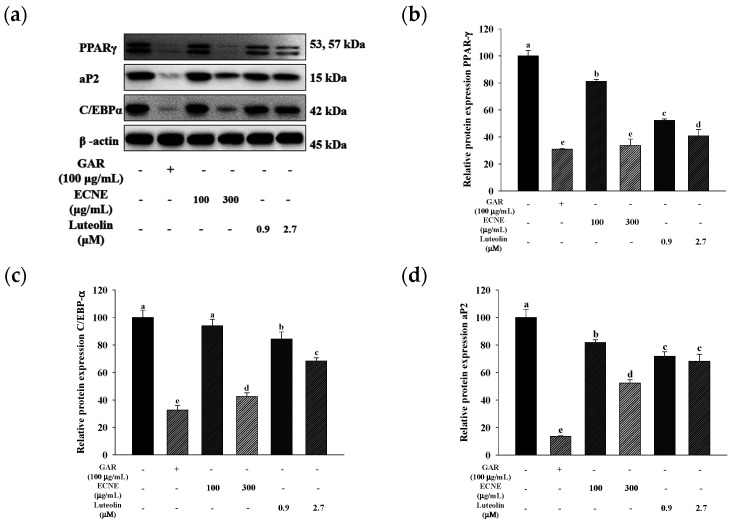
Effects of ECNE and luteolin on the expression of adipogenic transcription factors in differentiated 3T3-L1 adipocytes. (**a**) Representative Western blot images of PPARγ, C/EBPα, aP2, and β-actin. (**b**) Relative protein expression of PPARγ. (**c**) Relative protein expression of C/EBPα. (**d**) Relative protein expression of aP2. Protein expression levels were normalized to β-actin. *Garcinia cambogia* extract (GAR; 10 μg/mL) was used as a positive control. Data are presented as the mean ± SD of three independent experiments. Different lowercase letters indicate statistically significant differences among groups (*p* < 0.05) by Duncan’s multiple range test.

The protein expression of key adipogenic markers—PPARγ, C/EBPα, and adipocyte protein 2 (aP2)—was examined by Western blotting to elucidate the molecular mechanisms underlying the inhibitory effects of ECNE and luteolin on adipogenesis. Adipocyte differentiation is regulated by a coordinated transcriptional cascade in which PPARγ and C/EBPα function as central regulators driving the transition from preadipocytes to mature adipocytes, while aP2 serves as a downstream target of PPARγ and reflects terminal differentiation and lipid accumulation [28,29]. As shown in Figure 6a, the differentiated control group exhibited elevated expression levels of these adipogenic markers, indicating successful differentiation. Treatment with *Garcinia cambogia* extract (GAR), used as a positive control, markedly suppressed their expression. ECNE significantly downregulated the expression of PPARγ, C/EBPα, and aP2 in a concentration-dependent manner. Notably, at 300 μg/mL, ECNE reduced PPARγ expression to a level comparable to that observed in the GAR-treated group (Figure 6b). Densitometric analysis further confirmed that ECNE significantly decreased C/EBPα and aP2 expression relative to the differentiated control group (Figure 6c,d). Luteolin treatment also significantly reduced the expression of these adipogenic markers.

Collectively, these results indicate that ECNE inhibits adipocyte differentiation by suppressing the core adipogenic transcriptional program in 3T3-L1 cells.

### 3.3. Insulin-Sensitizing Effects of ECNE and Luteolin in Mature 3T3-L1 Adipocytes

#### 3.3.1. Cell Viability

**Figure 7:**
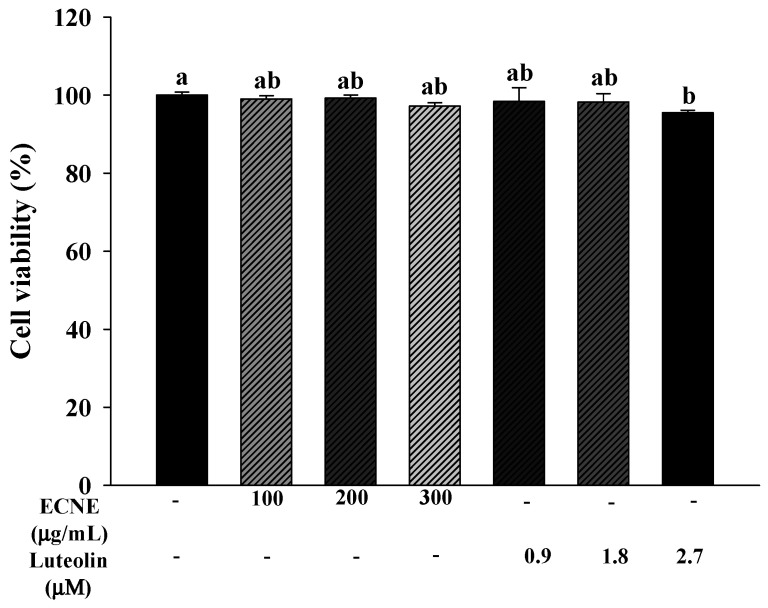
Effects of ECNE and luteolin on cell viability in mature 3T3-L1 adipocytes. Data are presented as the mean ± SD of three independent experiments. Different lowercase letters indicate statistically significant differences among groups (*p* < 0.05) by Duncan’s multiple range test.

The cytotoxicity of ECNE and luteolin in mature 3T3-L1 adipocytes was first evaluated by the XTT assay across the same concentration ranges used in the differentiation experiments. As shown in Figure 7, neither ECNE (100–300 μg/mL) nor luteolin (0.9–2.7 μM) reduced cell viability relative to the untreated control. Based on these results, ECNE (100 and 300 μg/mL) and luteolin (0.9 and 2.7 μM) were selected for the subsequent insulin sensitivity assays.

#### 3.3.2. Glucose Uptake

**Figure 8:**
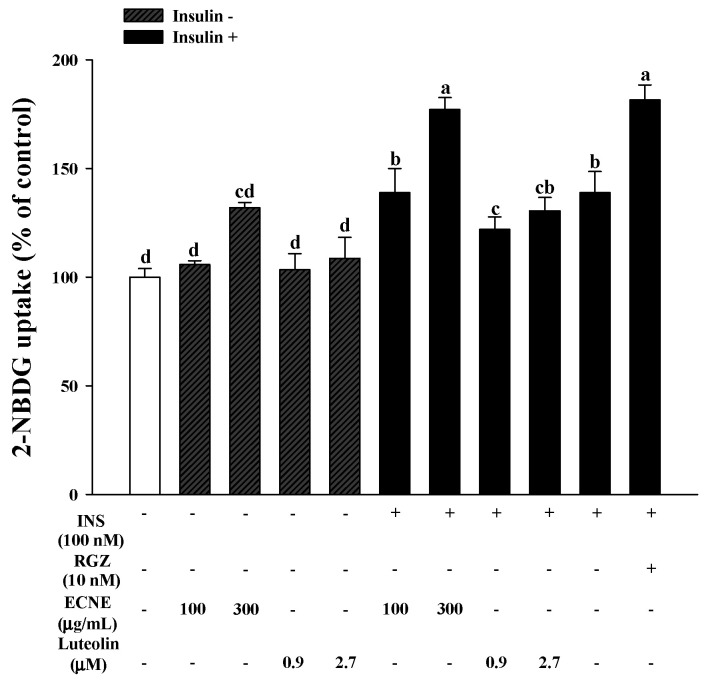
Effects of ECNE and luteolin on insulin-stimulated glucose uptake in mature 3T3-L1 adipocytes. Glucose uptake was measured under basal (insulin −) and insulin-stimulated (insulin +) conditions. Data are presented as the mean ± SD of three independent experiments. Different lowercase letters indicate statistically significant differences among groups (*p* < 0.05) by Duncan’s multiple range test.

To assess whether ECNE and luteolin enhance insulin sensitivity in fully differentiated adipocytes, glucose uptake was measured using the fluorescent glucose analog 2-NBDG. Rosiglitazone (RGZ), a PPARγ agonist known to improve adipocyte insulin responsiveness through enhanced insulin signaling and glucose transporter type 4 (GLUT4)-mediated glucose transport [30], was used as a positive control. As shown in Figure 8, basal glucose uptake (without insulin) was minimally affected by ECNE or luteolin. In contrast, insulin stimulation markedly increased glucose uptake in mature adipocytes. Under insulin-stimulated conditions, ECNE further enhanced glucose uptake in a concentration-dependent manner, with 300 μg/mL ECNE achieving levels comparable to those observed in the RGZ-treated group. Luteolin at 0.9 and 2.7 μM also significantly increased insulin-stimulated glucose uptake.

These findings indicate that ECNE and luteolin enhance insulin-stimulated glucose uptake in mature 3T3-L1 adipocytes, suggesting an improvement in insulin responsiveness. Collectively, these results highlight an additional metabolic effect of ECNE that extends beyond the inhibition of adipogenesis observed during adipocyte differentiation.

#### 3.3.3. Akt Phosphorylation

To investigate the molecular basis underlying the enhanced glucose uptake described above, the phosphorylation of Akt, a key regulator of insulin signaling, was analyzed by Western blotting in mature 3T3-L1 adipocytes. Upon insulin binding to its receptor, signaling is propagated through Akt phosphorylation, which mediates downstream processes including GLUT4 translocation and glucose uptake; conversely, impaired Akt activation is a well-established feature of adipocyte insulin resistance [31].

**Figure 9:**
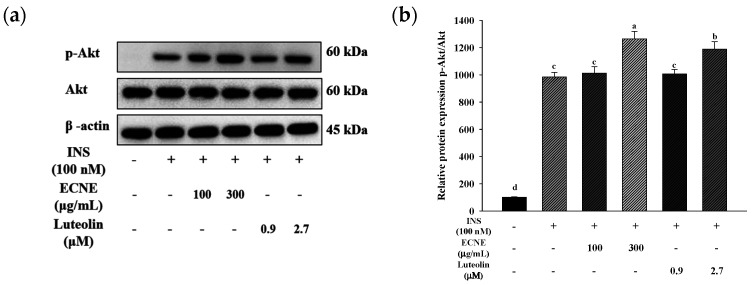
Effects of ECNE and luteolin on Akt phosphorylation in mature 3T3-L1 adipocytes. (**a**) Representative Western blot images of phospho-Akt (p-Akt), total Akt, and β-actin. (**b**) Relative protein expression of p-Akt normalized to total Akt. Data are presented as the mean ± SD of three independent experiments. Different lowercase letters indicate statistically significant differences among groups (*p* < 0.05) by Duncan’s multiple range test.

As shown in Figure 9a, insulin stimulation markedly increased Akt phosphorylation relative to the unstimulated control, while total Akt levels remained unchanged across all groups. Notably, ECNE further enhanced insulin-stimulated Akt phosphorylation in a concentration-dependent manner (Figure 9b), and luteolin treatment also increased this response.

These findings indicate that ECNE and luteolin enhance insulin signaling at the level of Akt activation, providing a mechanistic basis for the increased insulin-stimulated glucose uptake observed in Figure 8. Together, these results support their role as positive modulators of adipocyte insulin responsiveness.

### 3.4. Protective Effects of ECNE and Luteolin Under TNF-α-Induced Adipocyte Dysfunction

#### 3.4.1. Establishment of the TNF-α-Induced Adipocyte Dysfunction Model and Cytotoxicity Assessment

Adipocyte dysfunction in obesity is characterized by chronic low-grade inflammation and impaired insulin signaling [32]. To assess whether ECNE and luteolin maintain their effects under these conditions, mature 3T3-L1 adipocytes were exposed to TNF-α as an in vitro model of inflammation-associated adipocyte dysfunction.

**Figure 10:**
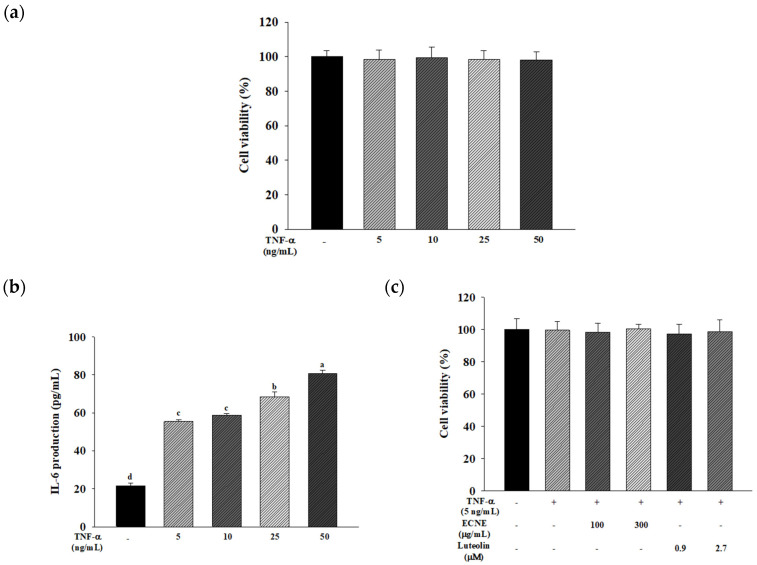
Establishment of the TNF-α-induced adipocyte dysfunction model in mature 3T3-L1 adipocytes. (**a**) Cell viability in response to TNF-α (0, 5, 10, 25, or 50 ng/mL). (**b**) IL-6 secretion in response to TNF-α (0, 5, 10, 25, or 50 ng/mL). (**c**) Cell viability of cells pretreated with ECNE (100 or 300 μg/mL) or luteolin (0.9 or 2.7 μM) followed by TNF-α (5 ng/mL). Data are presented as the mean ± SD of three independent experiments. Where shown, different lowercase letters indicate statistically significant differences among groups (*p* < 0.05) by Duncan’s multiple range test.

The cytotoxicity of TNF-α was first evaluated. Mature adipocytes (day 9) were treated with TNF-α at 0, 5, 10, 25, or 50 ng/mL for 24 h, and cell viability was assessed using the XTT assay. As shown in Figure 10a, TNF-α did not significantly affect cell viability at any tested concentration compared with the untreated control, indicating that concentrations up to 50 ng/mL are non-cytotoxic in mature 3T3-L1 adipocytes.

IL-6, a representative pro-inflammatory cytokine induced by TNF-α in mature adipocytes [33], was measured to determine the working concentration of TNF-α. As shown in Figure 10b, TNF-α increased IL-6 secretion in a dose-dependent manner, with a significant elevation observed from 5 ng/mL compared with the untreated control. Accordingly, 5 ng/mL TNF-α was selected for subsequent experiments based on its ability to induce a robust IL-6 response without cytotoxicity, representing the minimal effective concentration identified in our dose–response evaluation. While previous studies establishing TNF-α-induced models of inflammation and insulin resistance in 3T3-L1 adipocytes have typically employed concentrations ranging from 10 to 25 ng/mL [14,33], the present study adopted a milder concentration determined experimentally through systematic assessment of cytotoxicity and inflammatory cytokine secretion, allowing the inflammatory phenotype to be induced while minimizing excessive cellular stress.

Finally, the cytotoxicity of ECNE and luteolin in the presence of TNF-α was evaluated. Mature adipocytes were pretreated with ECNE (100 and 300 μg/mL) or luteolin (0.9 and 2.7 μM) for 24 h, followed by TNF-α (5 ng/mL) for an additional 24 h. As shown in Figure 10c, neither ECNE nor luteolin affected cell viability under these conditions, confirming their suitability for subsequent functional assays.

#### 3.4.2. Cytokine Production

**Figure 11:**
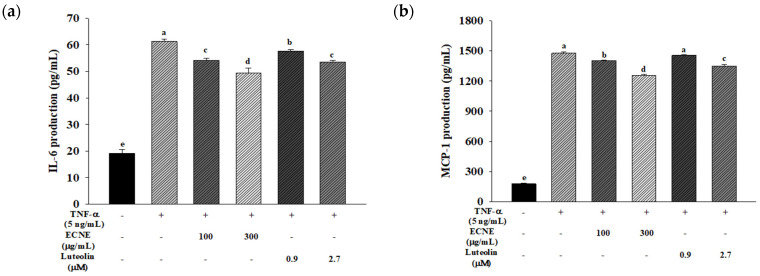
Effects of ECNE and luteolin on pro-inflammatory cytokine production in TNF-α-treated mature 3T3-L1 adipocytes. (**a**) IL-6. (**b**) MCP-1. Data are presented as the mean ± SD of three independent experiments. Different lowercase letters indicate statistically significant differences among groups (*p* < 0.05) by Duncan’s multiple range test.

Using the established TNF-α-induced inflammatory model, the effects of ECNE and luteolin on inflammatory responses were evaluated by measuring IL-6 and MCP-1 secretion. As shown in Figure 11, TNF-α (5 ng/mL) markedly increased IL-6 and MCP-1 levels compared with the untreated control, with approximately 3.2-fold and 8.3-fold elevations, respectively. Pretreatment with ECNE significantly attenuated TNF-α-induced IL-6 secretion in a concentration-dependent manner, with reductions of approximately 12% and 19% at 100 and 300 μg/mL, respectively. Luteolin also reduced IL-6 levels by approximately 6% and 13% at 0.9 and 2.7 μM, respectively. A similar trend was observed for MCP-1: ECNE reduced MCP-1 secretion by approximately 5% and 15% at 100 and 300 μg/mL, respectively, while luteolin significantly suppressed MCP-1 at 2.7 μM, whereas the lower concentration (0.9 μM) did not reach statistical significance.

IL-6 is known to impair insulin signaling [34], whereas MCP-1 promotes monocyte recruitment and amplifies adipose tissue inflammation [35]. Therefore, the simultaneous suppression of both cytokines by ECNE and luteolin suggests their potential to mitigate the inflammatory component of obesity-related adipocyte dysfunction, which is closely linked to the development of insulin resistance.

#### 3.4.3. Glucose Uptake

**Figure 12:**
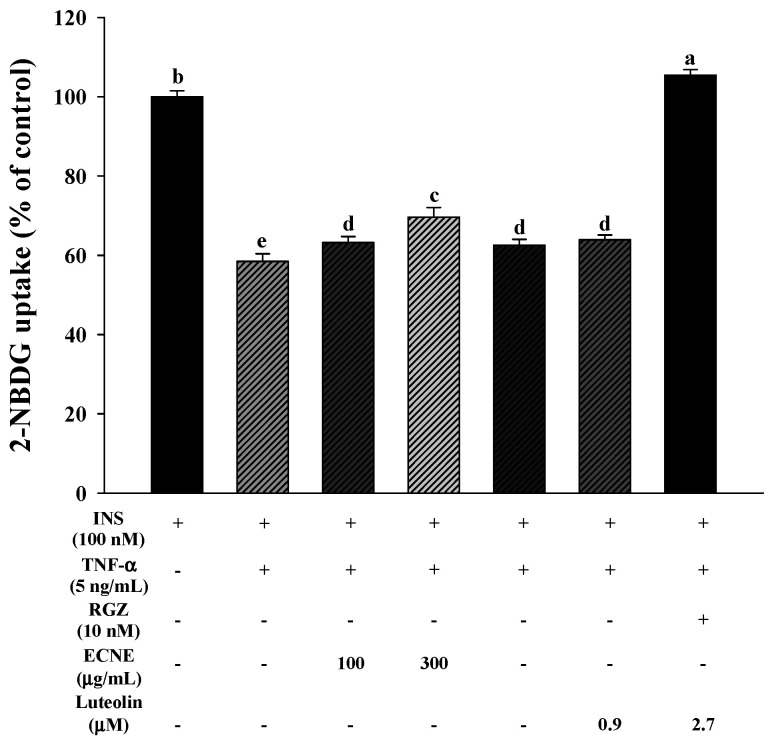
Effects of ECNE and luteolin on insulin-stimulated glucose uptake in TNF-α-treated mature 3T3-L1 adipocytes. Glucose uptake was measured using a 2-NBDG assay in cells stimulated with insulin (100 nM). RGZ (10 nM) was used as a positive control. Data are presented as the mean ± SD of three independent experiments. Different lowercase letters indicate statistically significant differences among groups (*p* < 0.05) by Duncan’s multiple range test.

To determine whether ECNE and luteolin restore insulin responsiveness under TNF-α-induced insulin resistance, insulin-stimulated glucose uptake was measured in mature 3T3-L1 adipocytes. RGZ was used as a positive control. As shown in Figure 12, TNF-α (5 ng/mL) reduced insulin-stimulated glucose uptake to approximately 58% of the untreated control, confirming the induction of an insulin-resistant state. TNF-α is known to impair insulin signaling and reduce glucose uptake in adipocytes [36], and the observed decrease is consistent with this established mechanism. Pretreatment with ECNE significantly attenuated the TNF-α-induced reduction in glucose uptake in a concentration-dependent manner, increasing uptake to approximately 63% and 70% of the control at 100 and 300 μg/mL, respectively. Luteolin also improved glucose uptake, restoring it to approximately 63% and 64% of the control at 0.9 and 2.7 μM, respectively. Notably, 300 μg/mL ECNE produced a greater recovery than 2.7 μM luteolin at corresponding luteolin-equivalent concentrations, suggesting that additional constituents contribute to the observed effect.

These findings indicate that ECNE and luteolin partially restore insulin-stimulated glucose uptake under TNF-α-induced insulin resistance.

#### 3.4.4. Akt Phosphorylation

**Figure 13:**
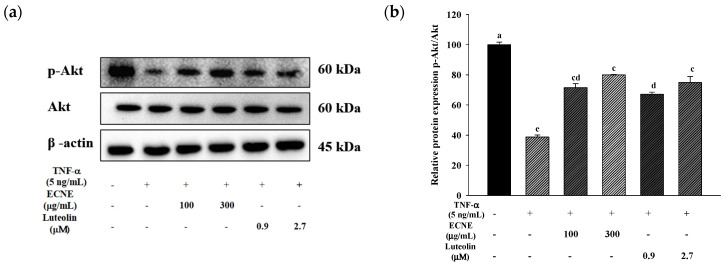
Effects of ECNE and luteolin on Akt phosphorylation in TNF-α-treated mature 3T3-L1 adipocytes. (**a**) Representative Western blot images of phospho-Akt (p-Akt), total Akt, and β-actin. (**b**) Relative protein expression of p-Akt normalized to total Akt. Data are presented as the mean ± SD of three independent experiments. Different lowercase letters indicate statistically significant differences among groups (*p* < 0.05) by Duncan’s multiple range test.

To examine the molecular basis of the observed recovery of glucose uptake, Akt phosphorylation was analyzed by Western blotting in mature 3T3-L1 adipocytes under TNF-α-induced insulin resistance. As shown in Figure 13a, TNF-α markedly reduced insulin-stimulated Akt phosphorylation compared with the insulin-stimulated control, while total Akt levels remained unchanged across all groups, indicating impaired insulin signaling.

Pretreatment with ECNE partially restored Akt phosphorylation in a concentration-dependent manner, with a more pronounced effect at 300 μg/mL, as confirmed by densitometric analysis (Figure 13b). Luteolin also enhanced Akt phosphorylation at both 0.9 and 2.7 μM, although the magnitude of recovery was comparable to or slightly lower than that observed with ECNE at corresponding luteolin-equivalent concentrations.

These findings indicate that ECNE and luteolin restore insulin signaling at the level of Akt activation under TNF-α-induced insulin resistance, providing a mechanistic basis for the recovery of insulin-stimulated glucose uptake observed in Figure 12. Notably, whereas ECNE and luteolin enhanced Akt phosphorylation under basal conditions (Figure 9), they restored its activation under inflammatory conditions, demonstrating efficacy across both physiological and pathological states.

Although the suppression of PPARγ during adipogenic differentiation (Figure 6) and the use of rosiglitazone as a positive control in mature adipocytes (Figure 8 and Figure 12) may initially appear contradictory, these observations should be interpreted in a phase-dependent manner by distinguishing the differentiation and maturation stages of adipocytes. During the differentiation phase, ECNE downregulated the PPARγ-driven adipogenic transcriptional program, thereby suppressing adipocyte formation. In contrast, in mature adipocytes, ECNE appeared to improve insulin responsiveness through modulation of insulin signaling pathways, as evidenced by the recovery of Akt phosphorylation, suggesting that this effect may be mediated, at least in part, through mechanisms partly independent of PPARγ activation. Of note, TNF-α itself has been reported to suppress PPARγ expression and impair insulin responsiveness in mature adipocytes [37]. Therefore, rosiglitazone served as a logical positive control by restoring PPARγ activity under inflammatory conditions. Importantly, the present study did not directly evaluate PPARγ expression or transcriptional activity in mature adipocytes, and the involvement of PPARγ-dependent mechanisms cannot be completely excluded. Future studies using PPARγ antagonists (e.g., GW9662) or PPARγ knockdown approaches will be required to further clarify the relative contributions of PPARγ-dependent and independent pathways to the insulin-sensitizing effects of ECNE.

Pro-inflammatory cytokines such as TNF-α and IL-6 are well known to impair insulin signaling in adipocytes by inducing serine phosphorylation of IRS-1 through activation of stress kinases such as JNK and IKKβ, thereby attenuating downstream PI3K/Akt signaling and glucose uptake [38,39]. Based on this established inflammation–insulin resistance axis, the simultaneous attenuation of IL-6 and MCP-1 secretion together with the restoration of insulin-stimulated Akt phosphorylation and glucose uptake observed in the present study suggests that the anti-inflammatory and insulin-sensitizing effects of ECNE may be mechanistically interrelated, with suppression of inflammatory cytokines contributing to the preservation of insulin signaling. Nevertheless, the present study did not include causal interventions to directly demonstrate this relationship, and the relative contributions of inflammation-dependent and -independent mechanisms warrant further investigation in future studies.

## 4. Conclusions

This study demonstrates the anti-inflammatory, anti-obesity, and insulin-sensitizing effects of the 40% ethanol extract of *Chamaecrista nomame* (ECNE) and its marker compound luteolin in cellular models of inflammation and adipocyte dysfunction. ECNE and luteolin suppressed nitric oxide and pro-inflammatory cytokine production in LPS-stimulated RAW 264.7 macrophages through inhibition of NF-κB and MAPK signaling pathways. In 3T3-L1 adipocytes, both treatments reduced lipid accumulation and downregulated key adipogenic transcription factors during differentiation, while enhancing insulin-stimulated glucose uptake and Akt phosphorylation in mature adipocytes. Importantly, these effects were consistently observed across individual cellular models and were maintained under TNF-α-induced adipocyte dysfunction, where ECNE and luteolin attenuated IL-6 and MCP-1 secretion and partially restored insulin-stimulated glucose uptake and Akt activation. Across all experimental conditions, ECNE exhibited effects comparable to or greater than those of luteolin at equivalent concentrations, suggesting that additional bioactive constituents contribute to its overall activity.

The consistently stronger effects of ECNE compared with equivalent concentrations of luteolin observed across multiple cellular models suggest that additional phytochemical constituents contribute to the overall bioactivity of the extract. A recent phytochemical analysis of *C. nomame* identified at least 16 compounds, including multiple flavone, chalcone, aurone, chromone, anthraquinone, and other phenolic derivatives, in addition to luteolin [9]. Many of these compound classes have been independently reported to exhibit anti-inflammatory, antioxidant, or metabolic regulatory activities and may therefore contribute to the overall effects of ECNE through mechanisms complementary to or distinct from those of luteolin. However, the present study does not allow us to determine whether these constituents act in an additive or synergistic manner. Future bioactivity-guided fractionation studies, together with testing of individual or recombined compounds, will be required to clarify the relative contributions of luteolin and non-luteolin constituents to the biological activity of ECNE.

The concentrations of ECNE (100 and 300 μg/mL) and luteolin (0.9 and 2.7 μM) used in this study were selected based on cytotoxicity assessment and efficacy screening, with luteolin tested at concentrations corresponding to its HPLC-quantified content in ECNE. It should be noted that these in vitro concentrations do not directly reflect achievable plasma or tissue concentrations in vivo, as the bioavailability of polyphenolic compounds such as luteolin can be limited by absorption, metabolism, and systemic distribution [40]. Therefore, the present findings should be regarded as mechanistic in vitro evidence supporting the biological potential of ECNE rather than direct evidence of physiological efficacy.

Collectively, these findings suggest that ECNE may serve as a promising natural resource with multi-targeted potential against obesity-associated inflammation and insulin resistance, although additional validation is required before any therapeutic applicability can be established. Future in vivo studies using high-fat diet-induced obese animal models are warranted to confirm the anti-inflammatory, anti-obesity, and insulin-sensitizing effects of ECNE, to evaluate its bioavailability and dose–response relationships, and to elucidate the contribution of its diverse bioactive components beyond luteolin. Building upon such preclinical validation, well-designed clinical studies will ultimately be required to assess the safety, efficacy, and translational potential of ECNE in humans.

## Data Availability

The original contributions presented in this study are included in the article/Supplementary Material. Further inquiries can be directed to the corresponding authors.

## Supplementary Materials

The following supporting information can be downloaded at: https://www.mdpi.com/article/10.3390/foods15111858/s1, Figure S1: HPLC chromatograms and PDA spectra of (a) the luteolin standard and (b) the 40% ethanol extract of *Chamaecrista nomame* (Siebold) H. Ohashi (ECNE).

## Abbreviations

The following abbreviations are used in this manuscript:

2-NBDG	2-(N-(7-nitrobenz-2-oxa-1,3-diazol-4-yl)amino)-2-deoxyglucose
aP2	Adipocyte protein 2
BS	Bovine serum
BSA	Bovine serum albumin
CE	Catechin equivalents
C/EBP-α	CCAAT/enhancer-binding protein-α
DEX	Dexamethasone
DMEM	Dulbecco’s modified Eagle’s medium
DMSO	Dimethyl sulfoxide
ECL	Enhanced chemiluminescence
ECNE	40% ethanol extract of *Chamaecrista nomame*
ELISA	Enzyme-linked immunosorbent assay
ERK	Extracellular signal-regulated kinase
FBS	Fetal bovine serum
GAR	*Garcinia cambogia* extract
HPLC	High performance liquid chromatography
IBMX	3-Isobutyl-1-methylxanthine
IκBα	inhibitor of nuclear factor kappa-B alpha
IL-1β	Interleukin-1β
IL-6	Interleukin-6
JNK	c-Jun N-terminal kinase
MAPK	Mitogen-activated protein kinase
MCP-1	Monocyte chemoattractant protein-1
NF-κB	Nuclear factor-κB
NO	Nitric oxide
ORO	Oil Red O
P/S	Penicillin-streptomycin
PBS	Phosphate-buffered saline
PPARγ	Peroxisome proliferator-activated receptor γ
RGZ	Rosiglitazone
TNF-α	Tumor necrosis factor-α
XTT	2,3-Bis(2-methoxy-4-nitro-5-sulfophenyl)-2H-tetrazolium-5-carboxanilide
