# Supplementary material for: Anti-Inflammatory, Anti-Obesity, and Insulin-Sensitizing Effects of Chamaecrista nomame (Siebold) H. Ohashi Extract in Cellular Models, Including TNF-α-Induced Adipocyte Dysfunction
Source: Foods. 2026 May 24;15(11):1858. doi: 10.3390/foods15111858 (PMC13257181; doi:10.3390/foods15111858)
Supplement: Supplementary file 1 [file foods-15-01858-s001.zip › foods-4315849-supplementary.pdf]

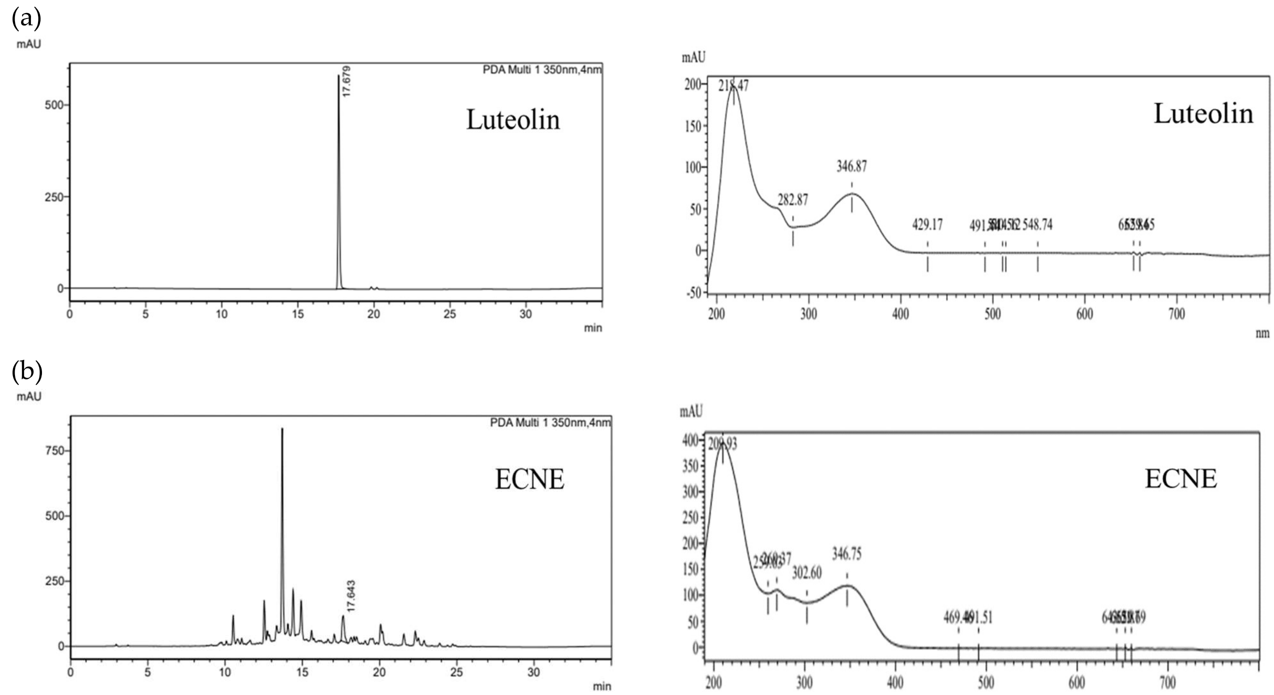

Figure S1. HPLC chromatograms and PDA spectra of (a) the luteolin standard and (b) the 40% ethanol extract of *Chamaecrista nomame* (Siebold) H. Ohashi (ECNE)
